# Analyzing surface EMG signals to determine relationship between jaw imbalance and arm strength loss

**DOI:** 10.1186/1475-925X-11-55

**Published:** 2012-08-22

**Authors:** Khoa Truong Quang Dang, Hoa Le Minh, Hai Nguyen Thanh, Toi Vo Van

**Affiliations:** 1 Biomedical Engineering Department, International University of Vietnam National Universities in Ho Chi Minh City, Quarter 6, Linh Trung Ward, Thu Duc District, Ho Chi Minh City, Vietnam

**Keywords:** Electromyography (EMG), Jaw imbalance, Arm strength, Integrated EMG, Higuchi fractal dimension

## Abstract

**Background:**

This study investigated the relationship between dental occlusion and arm strength; in particular, the imbalance in the jaw can cause loss in arm strength phenomenon. One of the goals of this study was to record the maximum forces that the subjects can resist against the pull-down force on their hands while biting a spacer of adjustable height on the right or left side of the jaw. Then EMG measurement was used to determine the EMG-Force relationship of the jaw, neck and arms muscles. This gave us useful insights on the arms strength loss due to the biomechanical effects of the imbalance in the jaw mechanism.

**Methods:**

In this study to determine the effects of the imbalance in the jaw to the strength of the arms, we conducted experiments with a pool of 20 healthy subjects of both genders. The subjects were asked to resist a pull down force applied on the contralateral arm while biting on a firm spacer using one side of the jaw. Four different muscles – masseter muscles, deltoid muscles, bicep muscles and trapezoid muscles – were involved. Integrated EMG (iEMG) and Higuchi fractal dimension (HFD) were used to analyze the EMG signals.

**Results:**

The results showed that (1) Imbalance in the jaw causes loss of arm strength contra-laterally; (2) The loss is approximately a linear function of the height of the spacers. Moreover, the iEMG showed the intensity of muscle activities decreased when the degrees of jaw imbalance increased (spacer thickness increased). In addition, the tendency of Higuchi fractal dimension decreased for all muscles.

**Conclusions:**

This finding indicates that muscle fatigue and the decrease in muscle contraction level leads to the loss of arm strength.

## Background

The relationship between the jaw muscles and other skeletal muscles are very important in explaining how and why the imbalance in the jaw causes loss in arm strength. Moreover, understanding this mechanism can bring many benefits for sports medicine by helping trainers design better mouth guards for athletes to improve their performance.

It has been reported that there is a strong relationship between the oral maxillofacial muscle, temporomandibular joint, and neck and arm muscles. Linderholm et al. [1] who investigated the relationship between bite force magnitude and forces produced by several skeletal muscle groups in children suggested that there was a relationship between bite forces and other forces, and the maximal bite force correlated with elbow flexion force and hand-grip force. Raadsheer et al. [2] found that the size of the jaw muscles was significantly related to the size of the limb muscles; however, maximal voluntary bite force moments were not significantly related to the moments of the arm flexion and leg extension forces.

Smith [3] performed a study to examine the effect of an increased vertical dimension of occlusion on isometric deltoid strength in 25 members of a professional football team with a variety of temporomandibular joint dysfunction, stomatognathic muscle and bite abnormalities. He concluded that there was a relationship between the jaws, posture, and the ability to the arm muscles to contract strongly. Forgione [4] analyzed the effect on isometric strength of biting on three intraoral devices and habitual occlusion using Nautilus lateral rise exercising device. They found that the average strength obtained with the elevated bite set to the functional criterion was significantly greater than in all other bite condition. It was concluded that a relationship does exist between bite and isometric strength.

In a study of finding a relationship between the height of bite plates and the strength of deltoid muscles, Chakfa et al. [5] performed experiments with 20 female subjects using the bite plates of adjusted heights of 2, 4, 6 and 12 mm. Each subject was seated on a dental chair with the arms extended to the side at the shoulder level and parallel to the floor. With or without introducing the bite plate in the subject’s mouth, the examiner applied a downward force on the wrist of the extended arm of the subject with a strain gauge while applying a stabilizing force on the contra lateral shoulder of the subject until the subject’s arm could no longer resist to the downward force. These researchers found that while increasing the height of the plate, the arm strength increased (from the strength without the plate of 6.9 kg on the right arm and 6.4 kg on the left arm) to a maximum (of 8.6 kg on either side of the arms) then decreased to 6.5 kg to the right and 6.3 kg to the left arm. Although these investigators did not indicate the height of the plate when the maximum strength occurred and simply reported that it depended on the subject, we deemed that they were either 4 or 8 mm.

In literature, the quantification of the jaw healthy and pain muscle activities during different jaw movements (opening, chewing, closing, etc.) were performed [6-9]. These studies showed a strong influence of the pain jaw muscles on the jaw movement patterns. Moreover, it has been showed that surface Electromyography (sEMG) technique is a good solution to measure and analyze, non-invasively, the jaw muscle activities.

In experiment, Ohtsuki [10] showed a decreasing voluntary isometric strength of the human arm under the condition of simultaneous bilateral exertion of contralateral corresponding muscles as compared to the unilateral condition. The decrease ratio of strength was 24.6 and 18.8% for the right and left for extension and 6.3 and 7.6% for the right and left for flexion. Integrated EMG showed the tendency comparable to the strength and high correlation was found between strength and integrated EMG. Recently, sEMG was used to predict the applied forces on human hands [11]. An artificial neural network was developed using frequency content of the sEMG signals. The investigators found a significant performance of the proposed model for predicting the loading forces based on the spectral features of the EMG signals.

**Figure 1:**
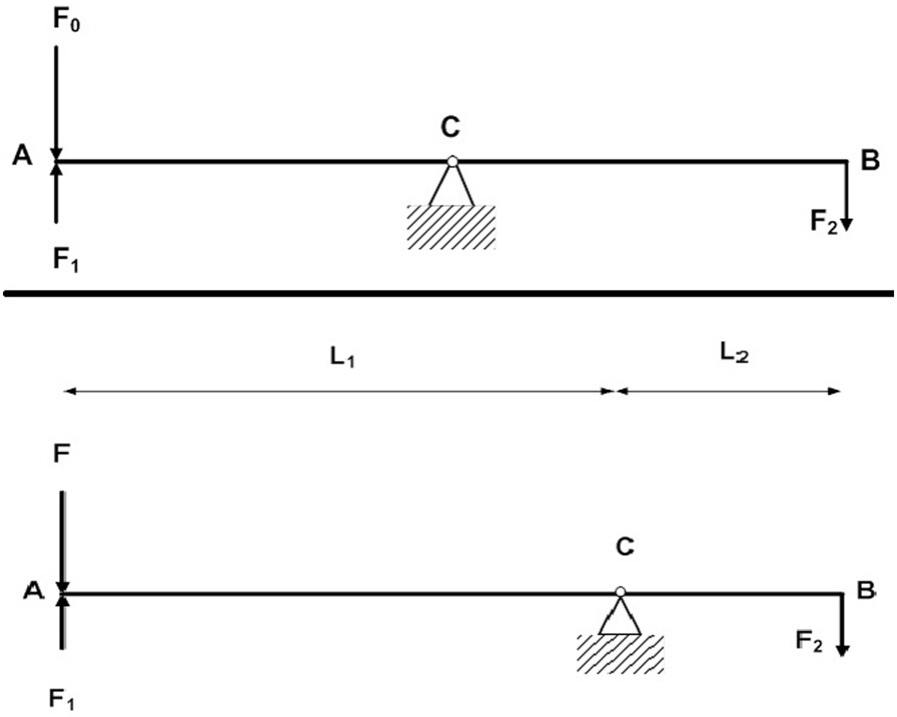
The feature of the system when the jaw balances and imbalances.

Our hypothesis is that there is a balance between the left and the right arm. It is represented as the mechanical system in which there is a fulcrum at the center of this system (Figure 1). When the imbalance of the jaw occurs, it makes the arm loss its strength. It means that the fulcrum move toward to the one side of the system. The advantage of this hypothesis is to establish the modeling to explain for this phenomenon. However, it cannot explain the physiology in side of this phenomenon.

In order to understand more about the bite and arm strength phenomenon, EMG was used to study. Our hypothesis is that whenever the jaw imbalance occurs, the EMG signal of the arm will be reduced. According to the best of our knowledge, there are no studies related directly to the quantification of muscle activities in relation to the bite and the isometric arms strength by measuring the arm strengths as well as the face and arm muscles activities using EMG technique. EMG is a powerful technique that was used to investigate the muscles activities when the firm spacer was placed in the subject’s mouth to create the imbalance of the jaw and the loss of arm strength. This gave us useful insights on the arms strength loss due to the biomechanical effects of the imbalance in the jaw mechanism.

In addition to understanding the EMG signal, integrated EMG and fractal dimension were used to analyze the signals. These analytical methods allow us to demonstrate the muscle activities and muscle fatigue in time domain and the complexity of the signal. Particularly, Higuchi fractal dimension is very useful in characterized EMG signals.

## Materials and methods

### Physical modeling

In this study to determine the effects of the imbalance in the jaw to the strength of the arms, we conducted experiments with a pool of 20 (age: 19.8 ± 0.9 years old) and healthy subjects of both genders. All participants were healthy and showed no musculoskeletal or neurological restrictions or diseases. They had a complete dentition, i.e. no premolar or molar was missing in a quadrant. There were no signs of severe malocclusions or facial malformations. All subjects were not on any medication and had no complain of any kinds of muscle pains throughout the experiments.

Before the experiments, each subject filled out a questionnaire, which was kept confidential and included patient’s identification, age and gender. The tenets of the Declaration of Helsinki were followed; local Institutional Review Board approved the study and informed consent was obtained for all subjects.

**Figure 2:**
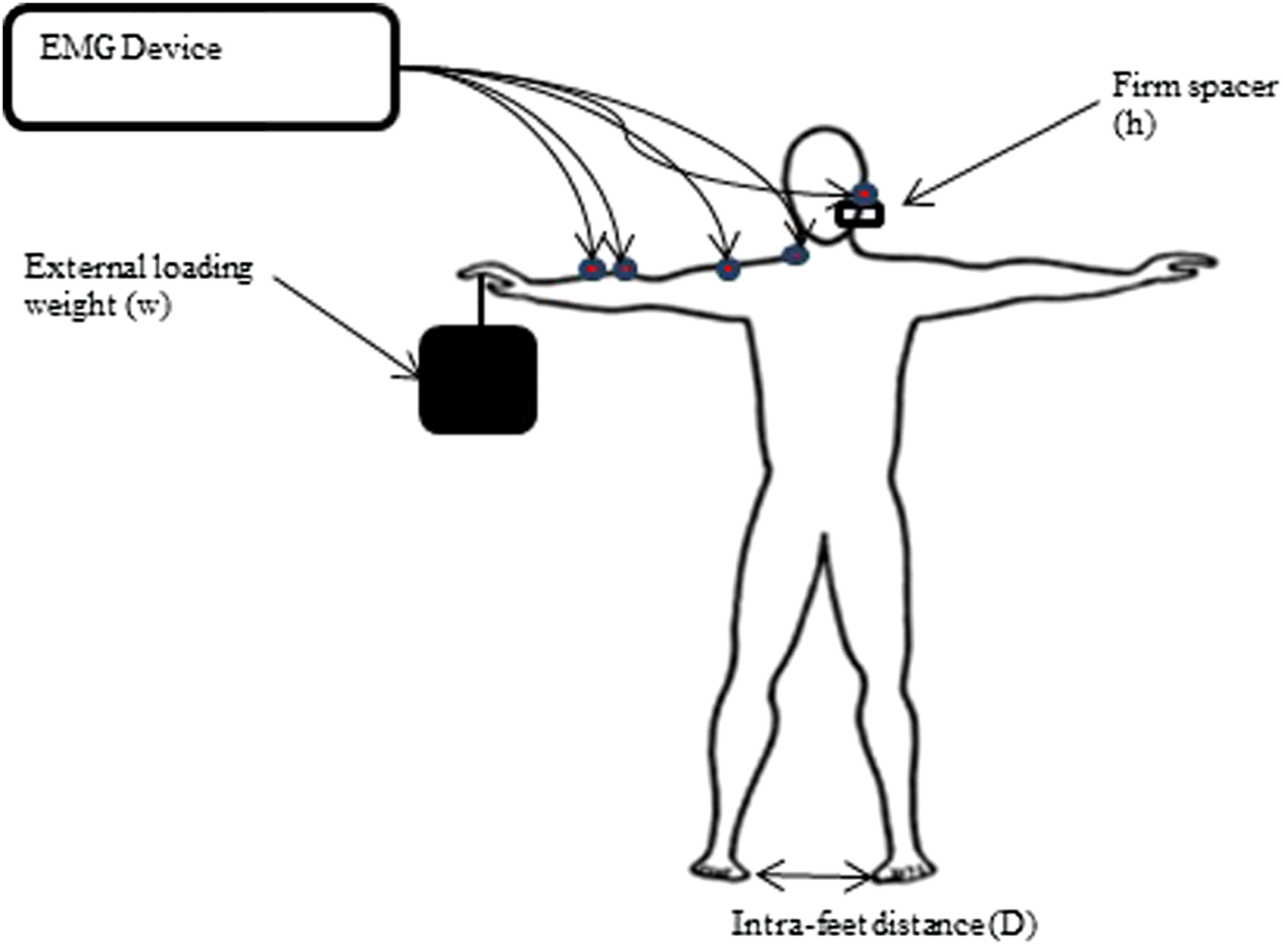
During the experiment, the subject stood with the arms and legs extended in frontal plan in such a way that the ratio D/H = 0.25.

The subjects were asked to stand with their arms and legs extended in frontal plan in such a way that the ratio subject intra-feet distance/ subject height equal 0.25 (Figure 2). This ratio was chosen based on the fact that many subjects felt comfortable and stable. The experimental protocol started with asking the subject hold the handle of a load which was at rest. Next at the warning signal the load was released at once; this generated a pull-down force on the subject’s arm. The subject must hold the load for a maximum of 15 seconds without moving his body. The pull-down force consisted of a load in kilogram that can be adjusted in the increment of 100 g and then converted into Newton. In these paradigms, the subject was asked to bite on a spacer using his premolar and molar teeth.

The subject held a spacer placed on one side of the jaw using premolar and molar teeth. The heights of the spacers were 0.6, 1.3 and 2.0 mm; however, they were applied in random order. Ten males (age 20 ± 0.9 years old) and 10 females (age: 19.8 ± 9.0 years old) participated.

**Figure 3:**
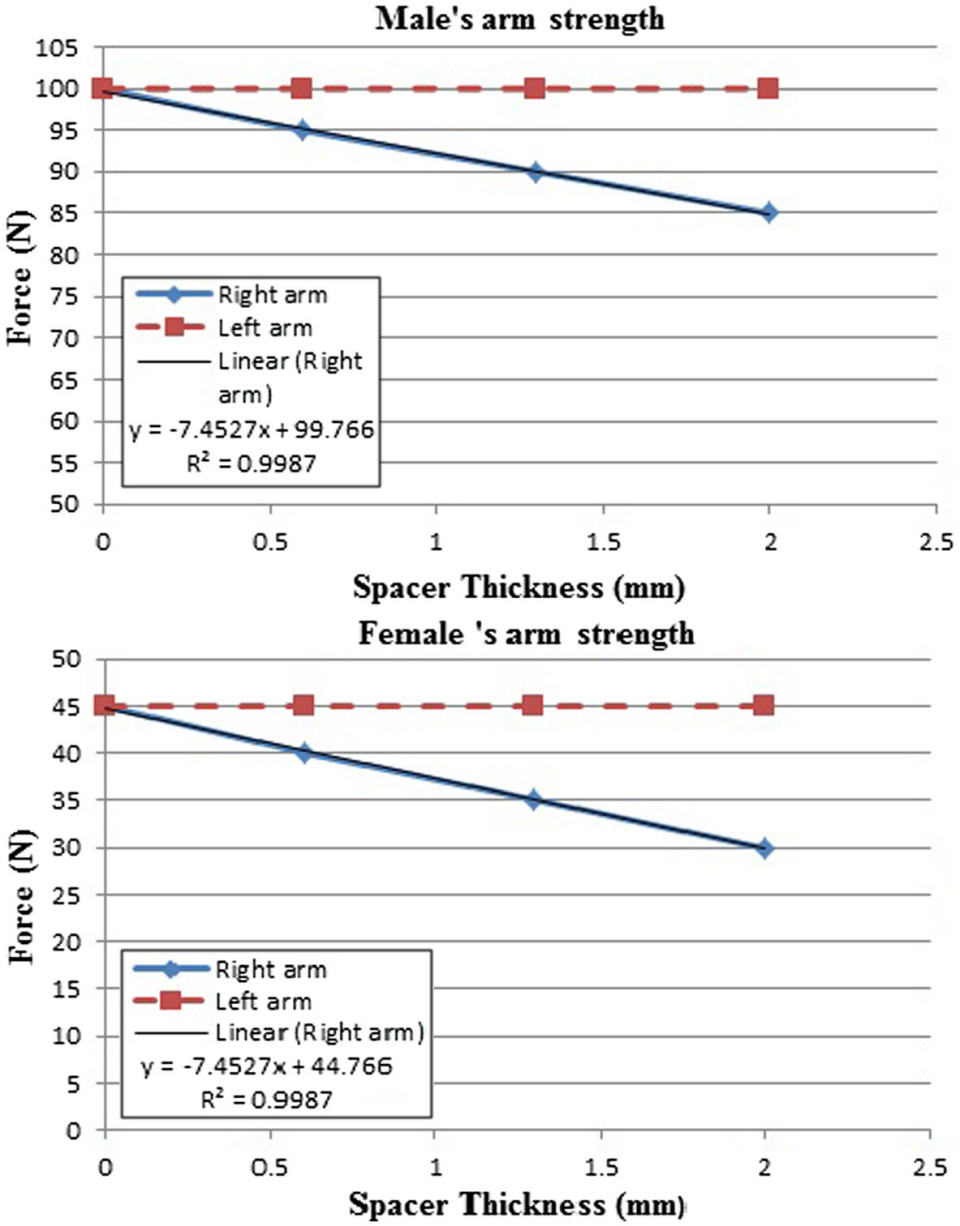
**Results of one male and one female subject.** We found that when the spacer was put in the left side of the jaw the right arm lost strength, while the left arm’s strength was intact.

We found that when the spacers were put in the left side of the jaw the loss of arm strength occurred on the right side of the arms and the strength of the arm on the same side intact. Similarly, when the spacers were put in the right side of the jaw the loss occurred on the left side of the arm while the strength of the arm on the same side was intact. This suggested that the bite imbalance causes a contralateral loss of strength in the arm (Figure 3).

In the process of understanding the origin of this phenomenon is muscular; we investigated the activities of muscles by using EMG. Ten male subjects participated in this investigation. The spacers had different heights of 2 mm and 3 mm and were applied in random order. The spacers were made out of a firm material to assume a good occlusal support. While the subject was biting on a spacer pull -down force was applied on the subject’s arm as aforementioned.

Myoelectric signals of jaw (masseter), neck (trapezoid) and arms (deltoid, brachiodiolis,) muscles were measured. The masseter was selected due to its main active role during biting at the temporomandibular (TM) joint. The concentric and eccentric contractions of the masseter create the biting motions as well as control the bite’s force. The trapezoid was selected due to its essential loading contribution in supporting the arm during persistent extension position. Arm extensors such as deltoid, brachiodiolis or extensor digitorum muscles were selected according to their role of support for maintaining the arm in extension under external loading conditions.

BIOPAC Systems Channel 1 was used to measure the activities of masseter muscle, channel 2 was used for trapezoid muscle, channel 3 was used for deltoid muscle, and channel 4 was used for branchi muscle. EMG (5- 500 Hz) was chosen to record EMG signal. The sampling rate was 1000/ second.The EMG measurement repeated twice for each experiment. The relaxation times within the experiments were 5 to 10 minutes. The recording time is 15 seconds in order to obtain the muscle fatigue [12,13]

Paradigm 1: The maximum force that the subject can resist in 15 seconds was recorded before taking EMG measurements. From the initial position (0 mm, Fmax-40 N), external loading weight was added incrementally with Fmax -20 N and Fmax. Check EMG signal quality and data storage.

Paradigm 2: Perform variation in spacer thickness from 2 mm to 3 mm and execute step 1 for Fmax and Fmax -20 N.

### Integrated EMG

To explain the fatigue of muscles by EMG signal in time domain, integrated EMG (iEMG) was calculated. Integrated EMG is the mathematical integration of rectified EMG signal. Simpson's rule was used to calculate iEMG**.** It is a method for numerical integration, the numerical approximation of definite integrals. Specifically, it is the following approximation:

(1) $$Y=\int_{a}^{b}f\left(x\right)dx\approx \frac{b-a}{6}\left[f\left(a\right)+4f\left(\frac{a+b}{2}\right)+f\left(b\right)\right]$$

In this formula (1), a and b are the unit spacing. f(a) and f(b) are the amplitudes of EMG signal at a and b. In fact, the iEMG was calculated as an approximation of the cumulative integral of the whole EMG signal amplitude during 15 seconds via the Simpson method with unit spacing.

**Figure 4:**
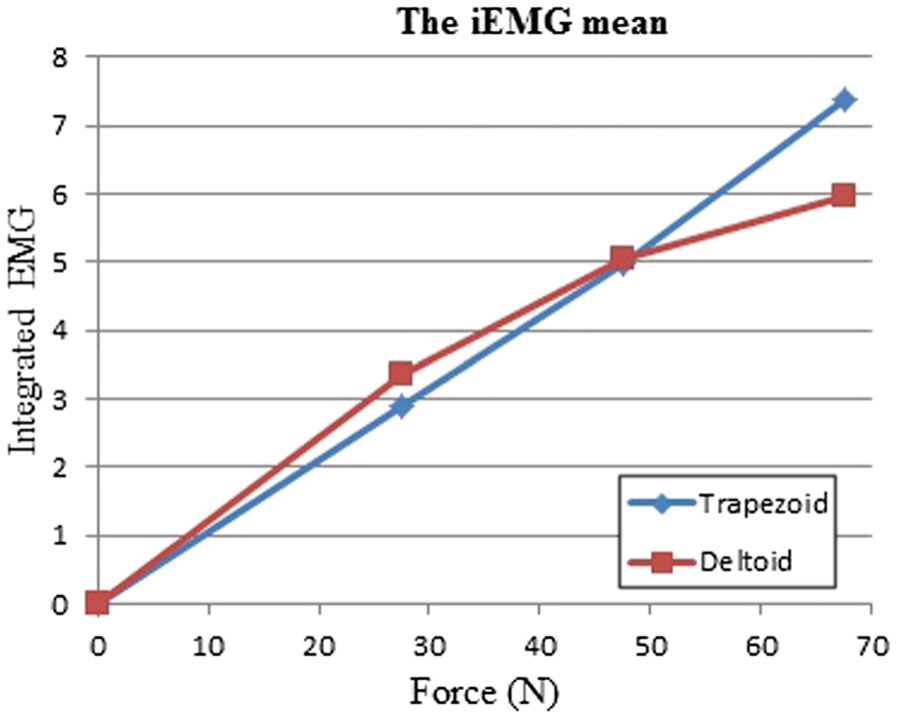
The iEMG mean of deltoid and trapeozoid muscles with variation in the external load without biting.

The iEMG mean of ten subjects significantly increased with respect to the increasing of external loading (Figure 4). With a bigger applied force, the muscles strongly contracted in respect to iEMG.

### Higuchi fractal dimension

Moreover, to understand the behavior of muscle through EMG signal, Higuchi fractal dimension was used. Higuchi is an efficient algorithm for measuring the fractal dimension of discrete time sequences [14]. Higuchi's algorithm calculates the fractal dimension from time series. Higuchi fractal dimension has already been used to analyze the complexity of surface EMG signal of the biceps [15].

Given a one dimensional time series X = x[1], x[2], …, x[N], the algorithm to compute the HFD can be described as follows [12]:

Form k new time series $X_{k}^{m}$ is defined by:

(2) $$X_{k}^{m}=\left\{x\left[m\right],x\left[m+k\right],x\left[m+2k\right],\ldots ,x\left[m+\mathrm{int}\left(\frac{N-m}{k}\right)\times k\right]\right\}$$

where k and m are integers, and int(·) is the integer part of ·. k indicates the discrete time interval between points, whereas m = 1, 2, …, k represents the initial time value.

The length of each new time series can be defined as follows:

(3) $$\overset{\mathrm{int}\left(\left(N-m\right)/k\right)}{i=1}\left|x\left[m+ik\right]-x\left[m+\left(i-1\right)\times k\right]\right|)\left[\left(N-1\right)/\left(\mathrm{int}\left(\left(N-m\right)/k\right)\times k\right)\right]\}$$

where N is length of the original time series X and (N − 1)/{int [(N − m)/k] × k} is a normalization factor.

Then, the length of the curve for the time interval k is defined as the average of the k values L(m,k), for m = 1, 2, …, k:

(4) $$L\left(k\right)=\frac{1}{k}\times \sum_{m=1}^{k}L\left(m,k\right)$$

Finally, when L(k) is plotted against 1/k on a double logarithmic scale, with k = 1, 2, …, k_max_, the data is expected to fall on a straight line, with a slope equals to the fractal of X. Thus, Higuchi fractal dimension is defined as the slope of the line that fits the pairs {ln[L(k)], ln(1/k)} in a least-squares sense. A value of k_max_ = 10 was chosen for our study.

## Results

### Physical modeling

Throughout the experiments to identify the loss of arm strength which was caused by the imbalance of the jaw, we found that:

1. There was a strong correlation between the loss of strength in the arms and the heights of the spacers (typically, the correlation coefficient was about 0.9). The correlation was estimated based on the results of 20 subjects (10 males and 10 females). The subject held a spacer on one side of the jaw using premolar and molar teeth. The pull-down force applied on one side of the arm in contralateral with the biting side. The correlation between the loss of arm strength and the thickness of spacer was calculated for each 40 cases (20 cases for each side). Then the average of these 40 cases was taken to get the final result.

2. The loss is inversely linear to the height of the spacers. Table 1 indicates the averages and standard deviations of the slope a and y-intercept b of male and female subjects when the spacers were placed in the left jaw and the measurements were done in the right jaw and the measurement were done in the left arm.

**Table 1:** Averages and standard deviations of the slope a and y-intercept b obtained with male and female subjects when the spacers were placed in the left jaw and the measurements were done in the right arms and when the spacers were placed in the right jaw and the measurement were done in the left arm

	**Right Arm**		**Left Arm**	
	**Male**	**Female**	**Male**	**Female**
**a[N/mm]**	−5.2 ± 1.9	−3.4 ± 2.6	−4.4 ± 2.0	−3.3 ± 1.7
**b[N]**	78.7 ± 19	37.9 ± 4.3	71.7 ± 16.7	35.2 ± 3.9

3. Within the male or female groups, the differences of the slopes a and the y- intercepts b obtained in the right arms and in the left arms were not statistically significant ( p> 0.05).

4. The difference between the slopes of male group and female group was not statistically significant (P > 0.05). However, the difference between the y-intercepts b of male group and female group was statistically significant (p < 0.05). This difference can be explained by the fact that male subjects were generally stronger than female subjects.

### Integrated EMG

The iEMG data was displayed by a box-plot diagram because it is convenient to represent the groups of data. It includes five number series; they are: minimum, lower quartile (Q1), median (Q2), upper quartile (Q3) and maximum. The spacing between different of the box indicate the degree of dispersion.

**Figure 5:**
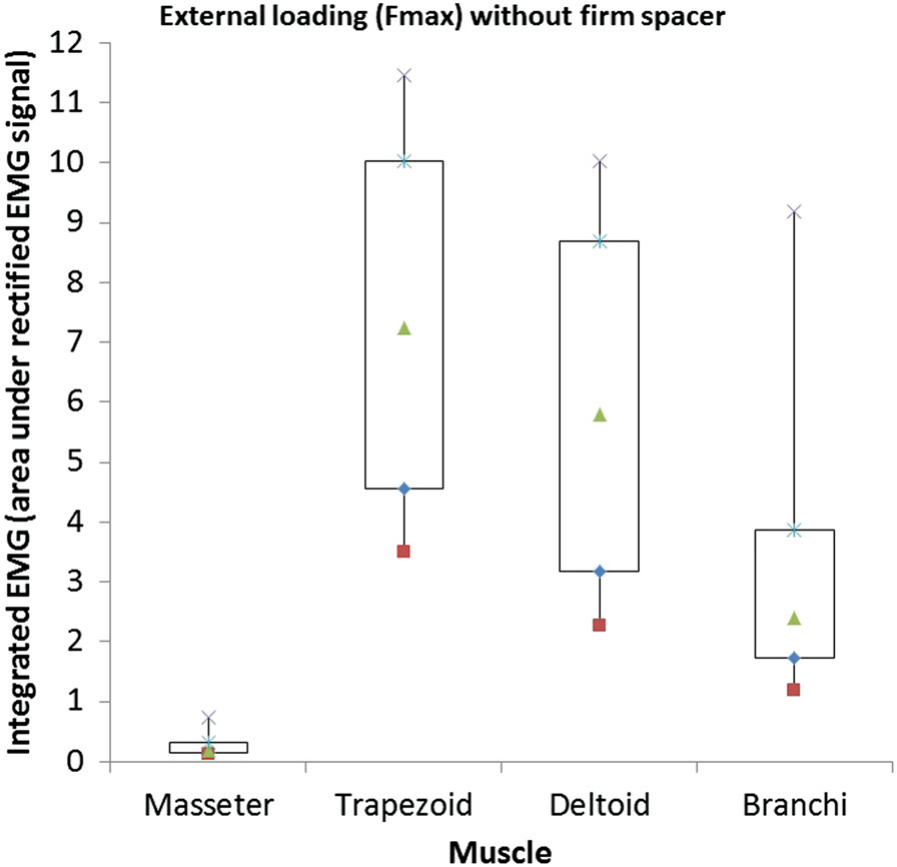
The iEMG of masseter, trapezoid, deltoid and branchi muscles when the applied force was Fmax without firm spacer.

When the subjects hold the load without a firm spacer (paradigm 1), integrated EMG (iEMG) of trapezoid, deltoid and branchi muscles are greater than the iEMG of masseter muscle. The results showed that the arm muscles are stronger than the jaw muscle (Figure 5). According to Table 2, the iEMG mean of the ten subjects, there are the increasing of iEMG of trapezoid, deltoid and branchi muscles when the external load increased, i.e.: mean of iEMG of trapezoid muscle is 2.89 when the external load is Fmax – 40 N, is 4.99 when the external load is Fmax – 20 N, is 7.39 when the external load is Fmax. In addition, the iEMG dispersion of the ten subjects also increases with the increasing of applied force. This means that the heavier external loading is the stronger arm muscle activities are.

**Table 2:** Mean ± SD of iEMG of ten subjects in paradigm 1

	**Fmax - 40 N**	**Fmax-20 N**	**Fmax**
Masseter	0.33 ± 0.22	0.30 ± 0.2	0.27 ± 0.19
Trapezoid	2.89 ± 1.05	4.99 ± 1.61	7.39 ± 3.14
Deltoid	3.35 ± 1.4	5.05 ± 2.28	5.96 ± 2.99
Branchi	1.25 ± 0.35	2.14 ± 1.19	3.62 ± 2.98

Table 3 shows the results of iEMG of masseter, trapezoid, deltoid and branchi muscles when the external load was Fmax-20 N without firm spacer, Fmax – 20 N with the 2 mm and 3 mm firm spacer in one side of the jaw. Mean of iEMG of trapezoid, deltoid muscles when the subjects bit on 3 mm thickness spacer are smaller than when the subjects bit on 2 mm firm spacer and without biting the spacer. In particular, mean ofi EMG of trapezoid when subjects bit on 3 mm firm spacer is 4.99 while it is 5.32 when subjects bit on 2 mm firm spacer and it is 5.68 as the normal occlusion. As a result, the loss of arm strength has occurred in this situation, especially for trapezoid and deltoid muscles.

**Table 3:** Mean ± SD of iEMG of ten subjects before and after biting when the applied force was Fmax –20 N

	**Fmax-20 without FS**	**Fmax-20 N with 2 mm FS**	**Fmax-20 with 3 mm FS**
Masseter	0.3 ± 0.2	1.15 ± 0.68	1.35 ± 0.93
Trapezoid	5.68 ± 1.74	5.32 ± 1.88	4.99 ± 1.6
Deltoid	5.19 ± 2.48	5.05 ± 2.3	4.85 ± 2.37
Branchi	2.14 ± 1.2	2.19 ± 0.86	2.35 ± 1.19

Table 4 shows the results of iEMG of masseter, trapezoid, deltoid and branchi muscles when the external load were Fmax with the 2 mm and 3 mm firm spacer inserted in one side of the jaw. The loss of arm strength has occurred which represent by the mean of iEMG. In the situation of the deltoid muscle, the iEMG when subjects bit on 3 mm firm spacer is 4.94 while it is 5.67 when subjects bit on 2 mm firm spacer.

**Table 4:** Mean ± SD of iEMG of ten subjects before and after biting when the applied force was Fmax

	**Fmax without FS**	**Fmax with 2 mm FS**	**Fmax with 3 mm FS**
Masseter	0.27 ± 0.19	1.25 ± 0.74	1.27 ± 0.75
Trapezoid	7.39 ± 3.14	7.06 ± 3.4	6.57 ± 3.44
Deltoid	5.98 ± 2.99	5.67 ± 2.92	4.94 ± 2.54
Branchi	3.62 ± 2.98	3.6 ± 2.88	3.14 ± 2.17

**Figure 6:**
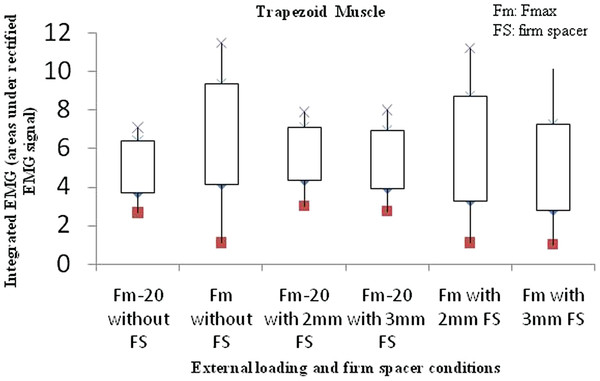
The iEMG of trapezoid muscle with variation in the external loading and firm spacer condition.

In particular, Figure 6 shows the iEMG results of the trapezoid muscles with and without placing thick spacer, iEMG with 2 mm and 3 mm firm spacer is smaller than iEMG without firm spacer, especially in the case of Fmax. The dispersion of iEMG within these ten subjects also decreases. This indicates the loss of arm strength due to the imbalance mechanism of the jaw.

**Figure 7:**
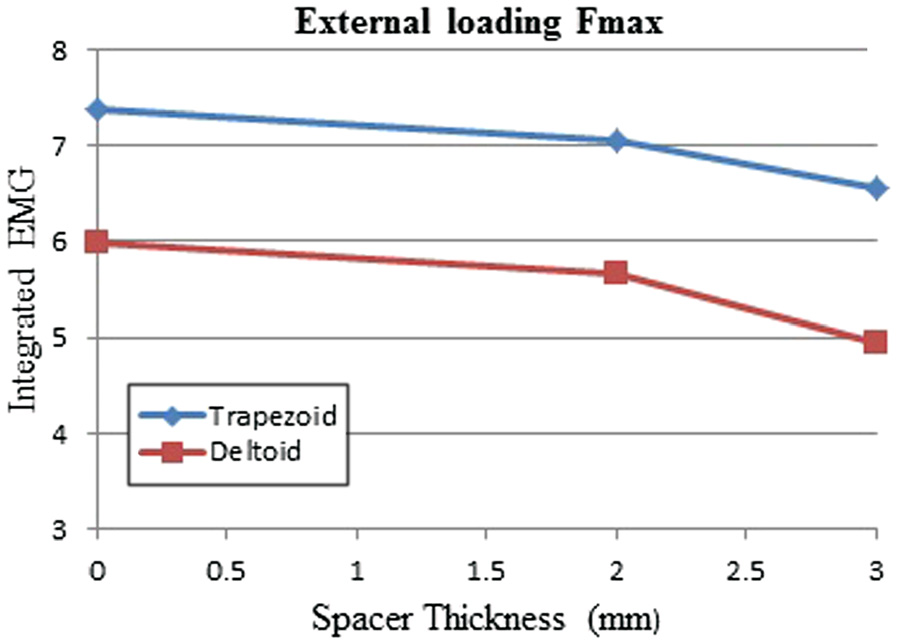
The iEMG mean of deltoid and trapezoid muscles with variation in the firm spacer when the applied force was Fmax.

In the experiment with biting variation in the thickness of the firm spacers, iEMG shows decreasing intensity of muscle activities (recruitment level of motor units). These results indicated that muscle fatigue occurred thus lowering the muscle contraction level, leading to loss in arm strength. The strength of the contralateral arm is reducing as the thickness of the spacer is increasing. Figure 7 shows the iEMG mean of trapezoid and deltoid muscles of ten subjects. It slightly decreases with respect to the increasing of firm spacer thickness.

### Higuchi fractal dimension

**Figure 8:**
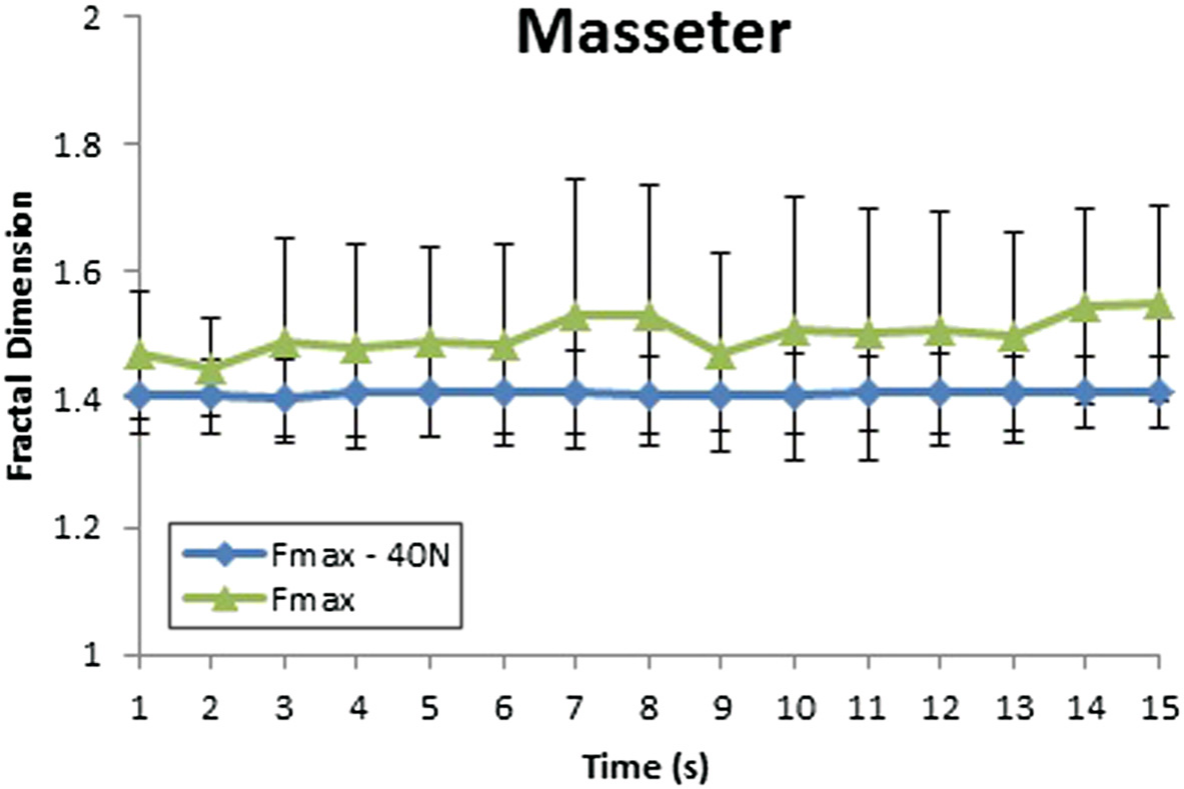
Fractal dimension of masseter muscle with variation in the external load without biting.

To understand the behavior of muscles activities during 15 seconds, Higuchi fractal dimension applied for every second. Figure 8 shows the fractal dimension of masseter muscle when different external forces were applied. There is no significantly difference in the fractal dimension of masseter muscles when the applied forces were Fmax – 40 N and Fmax – 20 N. Without biting, the level of fractal dimension of masseter muscle is around 1.4 because the masseter muscles did not activate. But the fractal dimension in case of the applied force was Fmax higher than the others. In this scenario, the masseter muscle was activated in the balance jaw condition to hold the heavy weight.

**Figure 9:**
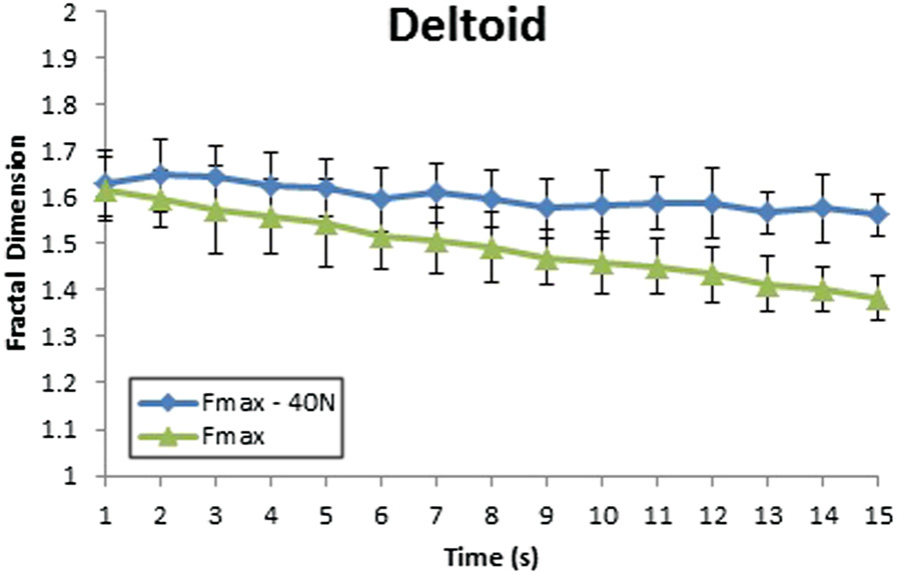
Fractal dimension of deltoid muscles with variation in the external load without biting.

Figure 9 shows the fractal dimension of deltoid muscle when the different external forces were applied. The level of fractal dimension also decreases with the increasing external loading. Particularly, the level of fractal dimension at Fmax – 40 N is stable at 1.6, but it slightly decreases from 1.6 to above 1.5 at Fmax – 20 N. It significantly decreases from 1.6 to 1.4 at Fmax. The decreasing tendency of deltoid muscle is 1/8 higher than the decreasing tendency of trapezoid muscle is 1/15. Therefore, deltoid and trapezoid play different role in supporting the arm’s strength.

The level of fractal dimension of branchi muscle is almost stable for different external loading. It’s quite different with deltoid and trapezoid muscles. Branchi muscle is in the lower part of the arm in comparison with deltoid and trapezoid, so it might not be affected by the changing of the applied force.

**Figure 10:**
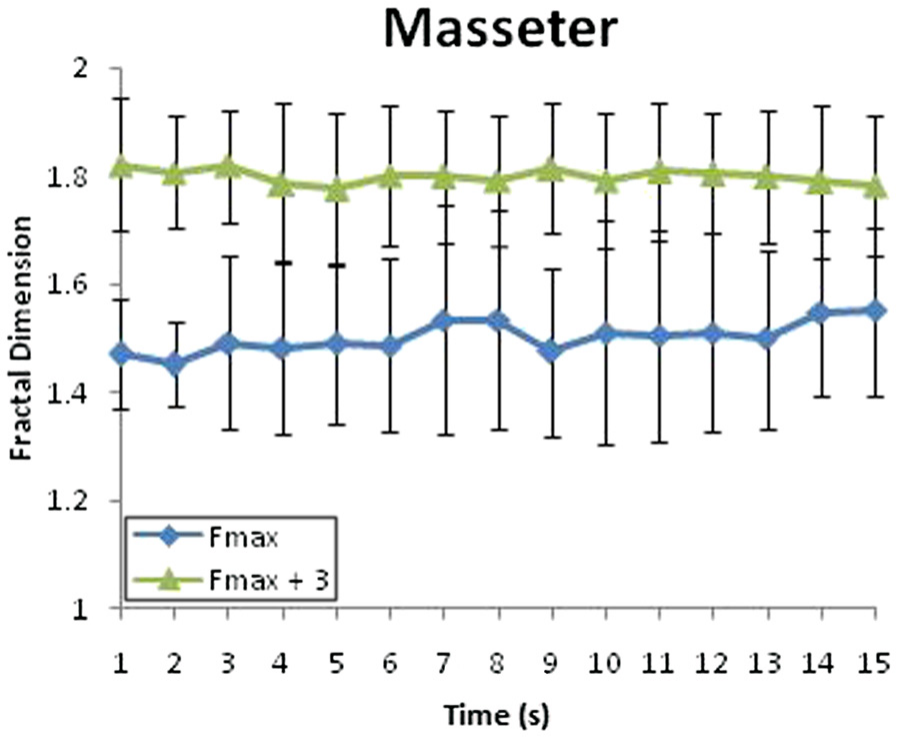
Fractal dimension of masseter muscles with variation in the firm spacer when the applied force was Fmax.

With the activities of masseter muscle before and after biting, the level of fractal dimension can be very well distinguished between two states (Figure 10). Specifically, before biting the spacer, the level of fractal dimension is around 1.5 but after biting level of fractal dimension is around 1.8. These levels of fractal dimension are stable which show the stable of biting force applied on the firm spacer. It also explains why there are no significantly difference between biting a 2 mm and 3 mm firm spacer.

**Figure 11:**
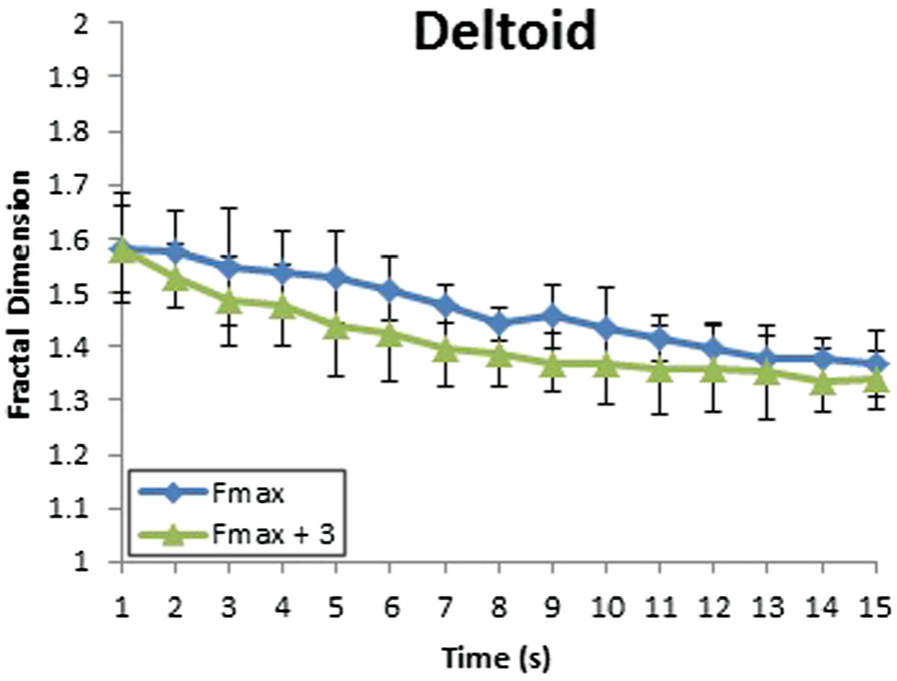
Fractal dimension of deltoid muscles with variation in the firm spacer when the applied force was Fmax.

The activities of deltoid muscle before and after biting show the differences of fractal dimension between before and after biting (Figure 11). The level of fractal dimension of Fmax + 3 is lower than the level of fractal dimension of Fmax. So the loss in arm strength according to the recording time occurs in this group of subjects. With the deltoid muscle, the level of fractal dimension indicates the differences in the complexity of muscle activities during 15 seconds. Muscle fatigue has occurred.

## Discussions

Indeed an imbalance of 0.5 mm in the jaw decreased 5% the arm strength and caused an imbalance in the two arms. Our finding confirmed the preliminary observations about the loss of arm strength due to the imbalance of the jaw. These results were identified based on the variation of iEMG and fractal dimension of four measuring muscles.

According to Smith [3], there was a relationship between the jaws, posture and the ability to the arm muscles to contract strongly. In comparison with this study, our investigation has successfully demonstrated that jaw imbalance cause loss in arm strength in contra-laterally. Moreover, deltoid and trapezoid muscles were involved the most in this phenomenon.

By using iEMG, our investigation also showed the correlation between jaw, arm muscles strength and iEMG as Ohtsuki [10] did. In the natural occlusion, the iEMG significantly increased with respect to the increasing of external loading. In the imbalanced occlusion, iEMG slightly decreases with respect to the increasing of firm spacer thickness which demonstrated the loss of arm strength.

Furthermore, EMG properties in temporal domain and fractal dimension allow us to have qualitative and quantitative evidence of the loss of arm strength during the bite. The experiments were repeated twice for each task to estimate the accuracy measurement.

## Conclusions

The results showed that (1) Imbalance bite induced contralateral loss of arm strength; (2) The loss was approximately a linear function of the height of the spacers; (3) The slopes of the linear function were similar in both arms and independent to the gender; and (4) the arm strength of male was higher than that of female subjects.

This study has successfully explained the loss of arm strength by the decreasing intensities of muscle contraction through iEMG property; and muscle fatigue through fractal dimension occurs during the bite under different external loading conditions.

Therefore, EMG is powerful tools to investigate the relationship between jaw imbalance and the loss of arm strength. The results from our research will shed the light for a better and deeper understanding of the mechanism of the loss of arm strength in the contralateral side during biting.

## Competing interests

The authors declare that they have no competing interests.

## Authors’ contribution

These authors contributed equally to this work. All authors read and approved the final manuscript.
